# Risk stratification by pre-operative cardiopulmonary exercise testing improves outcomes following elective abdominal aortic aneurysm surgery: a cohort study

**DOI:** 10.1186/2047-0525-2-10

**Published:** 2013-05-19

**Authors:** Stephen J Goodyear, Heng Yow, Mahmud Saedon, Joanna Shakespeare, Christopher E Hill, Duncan Watson, Colette Marshall, Asif Mahmood, Daniel Higman, Christopher HE Imray

**Affiliations:** 1 University Hospitals Coventry and Warwickshire NHS Trust, Clifford Bridge Road, Coventry CV2 2DX, UK; 2 Warwick Medical School, University of Warwick, Coventry CV4 7AL, UK

**Keywords:** AAA, Abdominal aortic aneurysm, CPET, Cardiopulmonary exercise test, Clinical outcomes

## Abstract

**Background:**

In 2009, the NHS evidence adoption center and National Institute for Health and Care Excellence (NICE) published a review of the use of endovascular aneurysm repair (EVAR) of abdominal aortic aneurysms (AAAs). They recommended the development of a risk-assessment tool to help identify AAA patients with greater or lesser risk of operative mortality and to contribute to mortality prediction.

A low anaerobic threshold (AT), which is a reliable, objective measure of pre-operative cardiorespiratory fitness, as determined by pre-operative cardiopulmonary exercise testing (CPET) is associated with poor surgical outcomes for major abdominal surgery. We aimed to assess the impact of a CPET-based risk-stratification strategy upon perioperative mortality, length of stay and non-operative costs for elective (open and endovascular) infra-renal AAA patients.

**Methods:**

A retrospective cohort study was undertaken. Pre-operative CPET-based selection for elective surgical intervention was introduced in 2007. An anonymized cohort of 230 consecutive infra-renal AAA patients (2007 to 2011) was studied. A historical control group of 128 consecutive infra-renal AAA patients (2003 to 2007) was identified for comparison.

Comparative analysis of demographic and outcome data for CPET-pass (AT ≥ 11 ml/kg/min), CPET-fail (AT < 11 ml/kg/min) and CPET-submaximal (no AT generated) subgroups with control subjects was performed. Primary outcomes included 30-day mortality, survival and length of stay (LOS); secondary outcomes were non-operative inpatient costs.

**Results:**

Of 230 subjects, 188 underwent CPET: CPET-pass *n* = 131, CPET-fail *n* = 35 and CPET-submaximal *n* = 22. When compared to the controls, CPET-pass patients exhibited reduced median total LOS (10 vs 13 days for open surgery, *n* = 74, *P* < 0.01 and 4 vs 6 days for EVAR, *n* = 29, *P* < 0.05), intensive therapy unit requirement (3 vs 4 days for open repair only, *P* < 0.001), non-operative costs (£5,387 vs £9,634 for open repair, *P* < 0.001) and perioperative mortality (2.7% vs 12.6% (odds ratio: 0.19) for open repair only, *P* < 0.05). CPET-stratified (open/endovascular) patients exhibited a mid-term survival benefit (*P* < 0.05).

**Conclusion:**

In this retrospective cohort study, a pre-operative AT > 11 ml/kg/min was associated with reduced perioperative mortality (open cases only), LOS, survival and inpatient costs (open and endovascular repair) for elective infra-renal AAA surgery.

## Background

*It is more important to know what sort of person has a disease than to know what sort of disease a person has* (Hippocrates, 460 to 370 BC).

Open abdominal aortic aneurysm (AAA) surgery places substantial metabolic demands upon patients during the perioperative period. These result from increased energy requirements necessary for wound healing [1], hemostasis, ventilation, significant intra-operative hemodynamic [2,3] and acid/base fluctuations in addition to the catecholamine stress response to surgery [4-6]. Failure of the cardiorespiratory system to meet these increased metabolic requirements of patients undergoing major abdominal surgery may lead to avoidable cardiorespiratory morbidity and mortality [7-10]. Aortic surgery is associated with a high (≥5%) combined incidence of cardiac death and non-fatal myocardial infarction [11]. An individual’s functional status has been shown to be reliably predictive of perioperative and long-term cardiac events following non-cardiac surgery [11], which can be derived from an assessment of their ability to perform activities of daily living [12,13]. Functional capacity (a numeric measure of functional status) can be expressed in metabolic equivalent (MET) levels; the oxygen consumption (VO_2_) of a 70-kg, 40-year-old man in a resting state is 3.5 ml/kg/min or 1 MET [11]. The American College of Cardiology and American Heart Association (ACC/AHA) guidelines for perioperative assessment states that patients able to demonstrate a functional capacity of 4 METS may safely proceed to surgery without further cardiac assessment [14]. This equates to the ability to climb a flight of stairs or run a short distance. However, subjective assessment of functional status by clinicians for patients undergoing AAA repair, can be easily confounded and lacks prognostic accuracy [15-17], identifying a potential role for objective testing.

Static pre-operative tests of cardiac function, such as resting left ventricular ejection fraction, correlate poorly with cardiorespiratory (physical) fitness [18,19], whilst dynamic tests such as dobutamine stress echocardiography and stress electrocardiogram (ECG) testing do not measure respiratory function and global oxygen delivery simultaneously. Cardiopulmonary exercise testing (CPET) allows the objective quantification of the level at which end-organ oxygen demand exceeds delivery [20] (the functional reserve) and may be safely performed in high-risk populations [7,15,21]. The transition point at which the production of CO_2_ exceeds VO_2_ is known as the anaerobic threshold (AT) and can be determined by gaseous exchange measurement during CPET [20]. More simply, the AT is the work rate at which an individual’s cardiorespiratory system fails to deliver sufficient oxygen to maintain aerobic respiration, mandating usage of an anaerobic substrate. AT is recognized as a reliable measure of pre-operative fitness in AAA patient populations [22].

Older *et al.* demonstrated that a critical AT ≥ 11 ml/kg/min for elderly subjects was associated with 0.8% perioperative mortality rate in major abdominal surgery, compared to 18% in individuals below this level [23]. In a further study, the same center identified a less intensive perioperative care requirement and reduced cardiovascular and all-cause mortality for elderly (major surgical) patients with AT ≥ 11, when compared to individuals below this threshold [24]. Additional work has shown anaerobic threshold >11 to be associated with fewer short-term complications and hence a shorter length of inpatient stay (LOS) following major abdominal surgery [25]. More contemporary evidence highlights other variables obtained during CPET to be at least as valuable (as AT) in predicting short- and mid-term outcomes in elective AAA surgery [7,26]. This is supported by a recent finding that AT < 10.2 ml/kg/min and peak oxygen consumption (VO_2_peak) < 15 ml/kg/min were associated with increased 30- and 90-day mortality following AAA surgery [27].

The aim of elective surgery for AAA is to prolong the survival of patients. However, there is an increasing awareness that many of these individuals have significant and potentially life-threatening cardiorespiratory co-morbidities [26]. Proactive screening for AAA reduces the prevalence of aneurysm-related mortality [28] and surgical intervention when the AAA ≥ 5.5 cm anterior-posterior (AP) diameter is appropriate [29] in units that have low perioperative complication rates. Large multicenter trials such as EVAR-1 have published 30-day mortality rates of 5.3% for open AAA repair [30]. However, the Vascular Society of Great Britain and Ireland’s (VSGBI) quality improvement framework (QIF) target of 3.5% by 2013 suggests that this could be improved further [31].

Pre-operative CPET was introduced for elective aneurysm surgery at University Hospitals Coventry and Warwickshire (UHCW) NHS Trust in 2007 in response to a 30-day mortality rate of 12.6% for open elective surgery, determined by internal audit and following an invited review by the VSGBI. These data reflected the hospitals’ all-comers policy to elective aneurysm surgery, offering operative repair to many individuals of equivocal cardiovascular health, who may have been declined intervention in vascular units with more stringent perioperative selection. The observed mortality rate for unselected patients prior to 2007 is comparable with the findings of Carlisle and Swart (30-day mortality rate: 9%) [26], who studied infra-renal AAA outcomes during a similar era. Whilst 12.6% 30-day mortality for unstratified (pre-CPET era) open AAA surgery appears unacceptably high at first glance, there is a significant moral dilemma to be addressed when consideration is given to the overall mortality rate for ruptured AAA, generally accepted as 90% [32]. Nevertheless, these mortality data fell beyond the established range quoted in the literature [33] and a trust-wide guideline of pre-operative CPET-stratification-based selection (for all elective AAA patients) was introduced, to facilitate ongoing AAA intervention at the established diameter of 5.5 cm, on a risk-benefit basis. Conservative management was offered to individuals considered to be at high risk of perioperative mortality following stratification, based upon an extrapolation of the 2007 Carlisle and Swart data [26].

This study aimed to assess the outcomes of pre-operative CPET-stratification on the duration of postoperative inpatient stay, intensive therapy unit (ITU) usage, end-organ support, 30-day mortality rates and longer-term survival following elective open and endovascular infra-renal AAA repair.

## Methods

This study is a retrospective, anonymized, single-center, cohort study performed at UHCW NHS Trust, a centralized vascular unit serving a population of 950,000. A review of the study proposal was undertaken by the trust’s Research and Development department; ethical approval was deemed unnecessary, based upon National Research Ethics Service guidance [34].

From November 2007, all patients considered for elective AAA repair (AAA ≥ 5.5 cm) surgery were recommended pre-operative CPET. An evidence-based minimum anaerobic threshold of 11.0 ml/kg/min was selected to identify individuals with adequate cardiopulmonary reserves who would be able to tolerate general anesthesia and open abdominal surgery, with acceptable perioperative mortality rates (the CPET-pass subgroup). These individuals were offered the option of endovascular aneurysm repair (EVAR), if anatomically applicable, or open AAA repair. Individuals attaining an AT < 11 ml/kg/min (the CPET-fail subgroup) were counseled regarding conservative management if EVAR (a less cardiovascularly challenging procedure) was not anatomically feasible. CPET-fail patients with unfavorable anatomy for infra-renal EVAR who requested open repair (rather than expectant management), were permitted to proceed following careful discussion and documentation of the perceived increased mortality risk. Individuals who were unable to demonstrate AT during CPET due to mechanical co-morbidities, suboptimal effort, suboptimal compliance with the investigation or ECG changes at minimal exertion (the CPET-submaximal subgroup) were managed as per the CPET-fail subgroup.

### Data collection and analysis

The data for 230 consecutive infra-renal AAA (≥5.5 cm AP diameter) patients considered for elective surgery between November 2007 and July 2011 (the CPET era) were studied. A control group of 128 consecutive individuals who underwent open or endovascular (infra-renal) AAA repair between January 2003 and October 2007 (the pre-CPET era) were identified for comparison. Patients diagnosed with thoracoabdominal or supra-renal aneurysms were excluded from the data collection, in addition to individuals who had undergone repairs of ruptured or urgent (symptomatic, non-ruptured) AAA.

Individuals were identified (with permission) by the Department of Clinical Coding using KMR1 diagnoses and procedures. To ensure completeness of data, results were cross-referenced with (computerized and written) operating theatre registries, the Dr Foster national outcomes database and the results of an internal audit of mortality/morbidity for elective AAA patients. Cardiopulmonary exercise testing data for CPET-era patients were collected (with full permission of the Department of Respiratory Physiology) from the UHCW CPET database.

Demographic and outcome data were identified by a systematic review of the hospitals’ Clinical Results Reporting System (CRRS) and the patient case notes. Data loss was minimized by cross-referencing, using anonymized patient identification (PID) numbers, with the ITU patient digital registry and the CPET database. Information relating to length of ITU stay and the number of end organs supported was obtained from the ITU patient digital registry. Mortality data were sourced (with permission) from CRRS, the hospitals’ Bereavement Services Department and by liaison with primary care providers. Survival was calculated from the date of intervention until death or censorship, in days. For individuals managed conservatively (CPET era), survival was calculated from the date of CPET until death or censorship. Survival could be observed for up to eight years in the control cohort and a maximum of four years for the CPET-era cohort, within the constraints of this study and this is regarded as mid-term survival. Tariffs for ITU and ward stays were obtained from the Department of Health Report 2010/2011 for UHCW via the Finance Section of the Information Services department. ITU cost data were calculated on an individual basis. A variable tariff applied, based upon the total number of end organs postoperatively supported (Table 1) multiplied by the duration of ITU stay. Ward costs were calculated by multiplication of a standard tariff (Table 1) by duration of ward stay. Financial analysis did not consider the costs of staff, equipment and consumables, which are essentially constant throughout NHS organizations offering similar interventions.

**Table 1:** Ward-based and critical care unit costs per 24 h stay

**Location**	**Discriminator**	**Cost per day (£)**
CPET	One-off tariff	150
Surgical ward	Influenceable costs	110
	Fully absorbed costs	250
ITU	0 organs supported	260
	1 organ supported	769
	2 organs supported	1106
	3 organs supported	1386
	4 organs supported	1511
	5 organs supported	1568
	6 organs supported	1638

### Surgical technique

All elective infra-renal AAA repairs were discussed and planned within a multidisciplinary team. Open aneurysm repairs were performed by a consultant vascular surgeon, or a consultant-supervised higher surgical trainee, using a transperitoneal inlay repair with knitted Dacron graft prostheses. Endovascular aneurysm repairs were planned and performed by a consultant vascular surgeon and consultant interventional radiologist. The EVAR devices used were the Cook Zenith® (Cook, Brisbane, Australia) endovascular system, Medtronic Endurant® (Medtronic, Minneapolis, MN, USA) and Lombard Aorfix™ (Lombard Medical, Oxfordshire, UK).

### Cardiopulmonary exercise testing

Prior to testing, the patient’s body mass index (BMI) was determined by measurement of height and weight. Resting spirometry was performed to measure forced expiratory volume per second (FEV_1_) and forced vital capacity (FVC), from which FEV_1_/FVC ratios were calculated. Predicted FEV_1_ and FVC values were derived as a function of age, height, ethnicity and gender (calculated by the CPET software – see below). FEV_1_ data were used to assess an individual’s maximum predicted ventilation. The patient’s weight and predicted maximum oxygen uptake (VO_2_max) were used to calculate the individually required work rate on the cycle ergometer. Patients were subsequently attached to a 12-lead ECG and a form-fitting face-mask connected to a metabolic cart with protocol specific software (Viasprint Ergometer and MasterScreen CPX software v5.21.0.60, CareFusion Corporation, CA, USA). Patients were initially made to pedal for an unloaded phase (work rate 0 W at 50 rpm for 3 min) followed by a ramped phase requiring a constant 70 rpm against increasing resistance until they reached their peak oxygen uptake (VO_2_peak). The test could be stopped at any point during the test protocol due to patient fatigue, presence of ischemic ECG changes, chest pain or if the maximum heart rate was achieved. The AT was derived using the V-slope method as described by Beaver *et al.*[35].

### Statistical analysis

All data were tabulated using a Microsoft Excel® spreadsheet (Microsoft, CA, USA) and statistical analyses performed using Graphpad Prism4® (Graphpad, La Jolla, CA, USA). The normality of data was assessed with the D’Agostino and Pearson omnibus normality test. Non-parametric unpaired data were analyzed using the Mann–Whitney *U* test or Kruskal–Wallace analysis of variance (ANOVA), whilst categorical variables were analyzed using the chi-squared test or Fisher’s exact test. Parametric data were assessed with a Student’s t-test or one-way ANOVA. Survival data were evaluated by Kaplan–Meier curves. A *P* value of less than 0.05 was considered significant.

## Results

Of 128 control subjects in the pre-CPET era, there were 103 (80.5%) open AAA repairs and 25 (19.5%) EVARs. Following introduction of CPET, 230 consecutive subjects with elective infra-renal AAA were studied. Operated cases included open repair in 100 (59.2%) patients and EVAR in 69 (40.8%), representing a significant increase in the proportion of endovascular cases (*P* < 0.001). A further 61 individuals did not have intervention.

**Figure 1:**
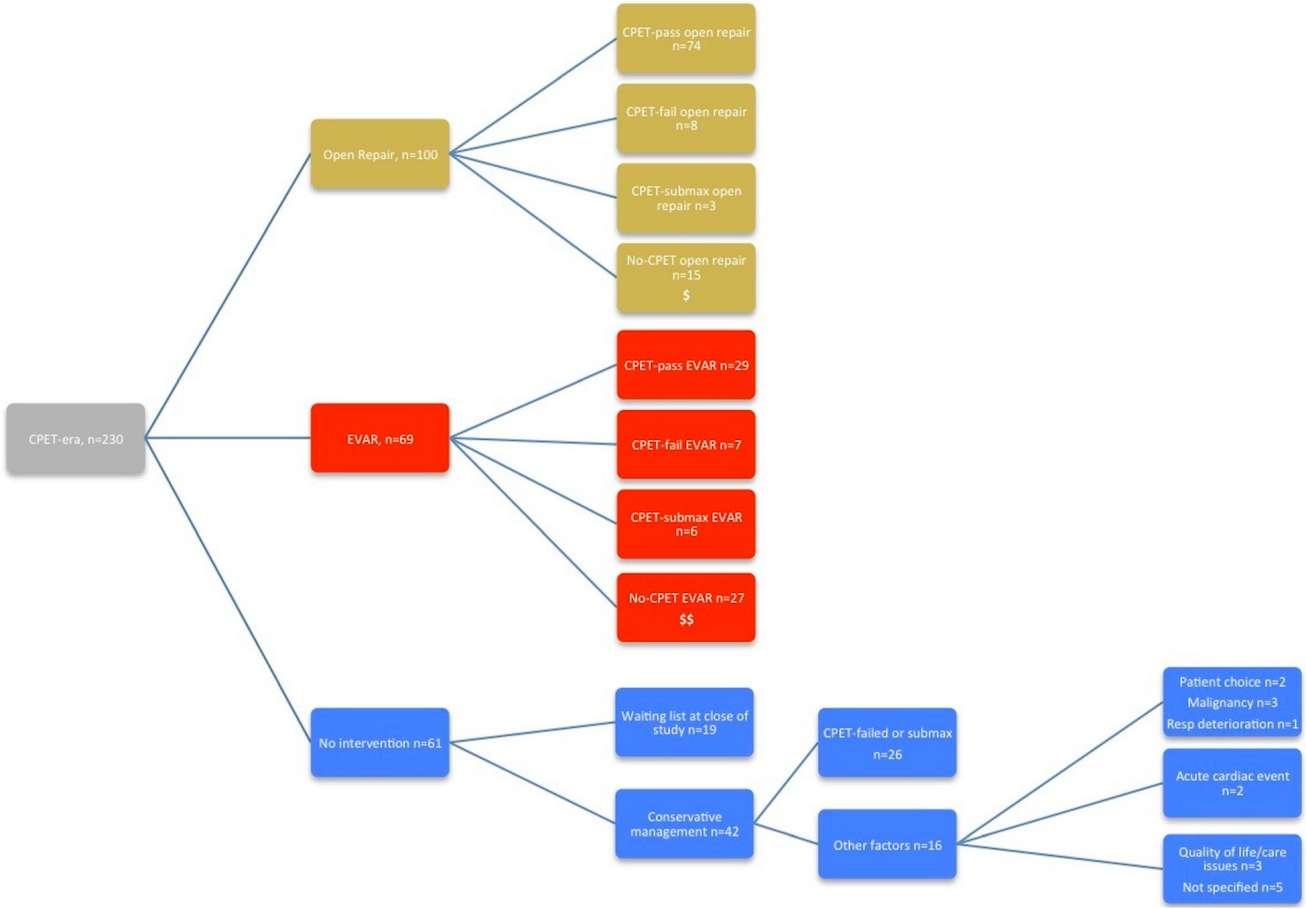
**A consort-type diagram representing the composition of the CPET-era cohort by management type and their subgroups by CPET stratification.** $ and $$ indicate the 42 individuals who were not referred for CPET within this cohort.

Composition of the CPET-era open surgery, EVAR and no intervention groups, with respect to CPET outcomes, are demonstrated in Figure 1.

### CPET subgroups

Of the 230 subjects identified in the CPET era, 188 underwent CPET. Tested individuals were stratified by their anaerobic threshold into three cohorts: CPET-pass (*n* = 131, AT ≥ 11.0 ml/kg/min), CPET-fail (*n* = 35, AT < 11.0) and CPET-submaximal (*n* = 22, unable to generate an AT). Of the patients, 42 were not referred for CPET (Figure 1).

Table 2 shows demographic data for pre-CPET era controls and CPET-era subgroups. Control subjects were of comparable age (median age: 74.0 years, 95% CI: 71.9 to 74.4) and equivalent aneurysm size (median aneurysm diameter: 6.3 cm, 95% CI: 6.5 to 6.9) to those in the CPET-stratified subgroups. BMI was infrequently recorded during the pre-CPET era rendering these data unsuitable for comparison. However, the median age was significantly higher in the CPET-submaximal group compared to CPET-pass patients (*P* < 0.01), the untested cohort (*P* < 0.01) and the pre-CPET cohort (*P* < 0.01). In addition, BMI was significantly lower in the CPET-pass cohort than the CPET-fail group (*P* < 0.05).

**Table 2:** Demographic data for CPET-era elective infra-renal AAA patients compared to pre-CPET era controls

	**Pre-CPET era (*n *** **= 128)**		**Elective AAA patients – CPET performed (*n *** **= 188)**						**No CPET (*n *** **= 42)**	
			**CPET-pass (*n *** **= 131)**		**CPET-fail (*n *** **= 35)**		**CPET-submaximal (*n *** **= 22)**			
	**Median**	**95% CI**	**Median**	**95% CI**	**Median**	**95% CI**	**Median**	**95% CI**	**Median**	**95% CI**
Age (years)	74.0 (**)	71.9 to 74.4	74.0 (**)	72.1 to 74.7	75	73.1 to 78.3	**80.5**	76.7 to 81.4	72.5 (**)	70.1 to 74.8
BMI (kg/m^2^)	N/A	N/A	27.3 (*)	26.8 to 28.2	**30.0**	27.6 to 31.4	27.6	25.7 to 31.3	N/A	N/A
Aneurysm size (cm)	6.3	6.5 to 6.9	6.1	6.2 to 6.6	6.1	6.0 to 6.7	6.3	6.0 to 6.9	5.9	5.9 to 6.5

*P < 0.05, **P < 0.01; significance when compared to the figure highlighted in bold for the given row.

### Length of inpatient stay

The median length of inpatient stay was significantly longer for open AAA surgery than EVAR in both the pre-CPET (open surgery: 13 days, 95% CI: 13.9 to 19.0; EVAR: 6 days, 95% CI: 4.3 to 8.3 days; *P* < 0.001) and post-CPET (open surgery: 10 days, 95% CI: 10.3 to 13.5; EVAR: 4 days, 95% CI: 4.6 to 6.7 days; *P* < 0.001) cohorts.

#### Open surgery

**Figure 2:**
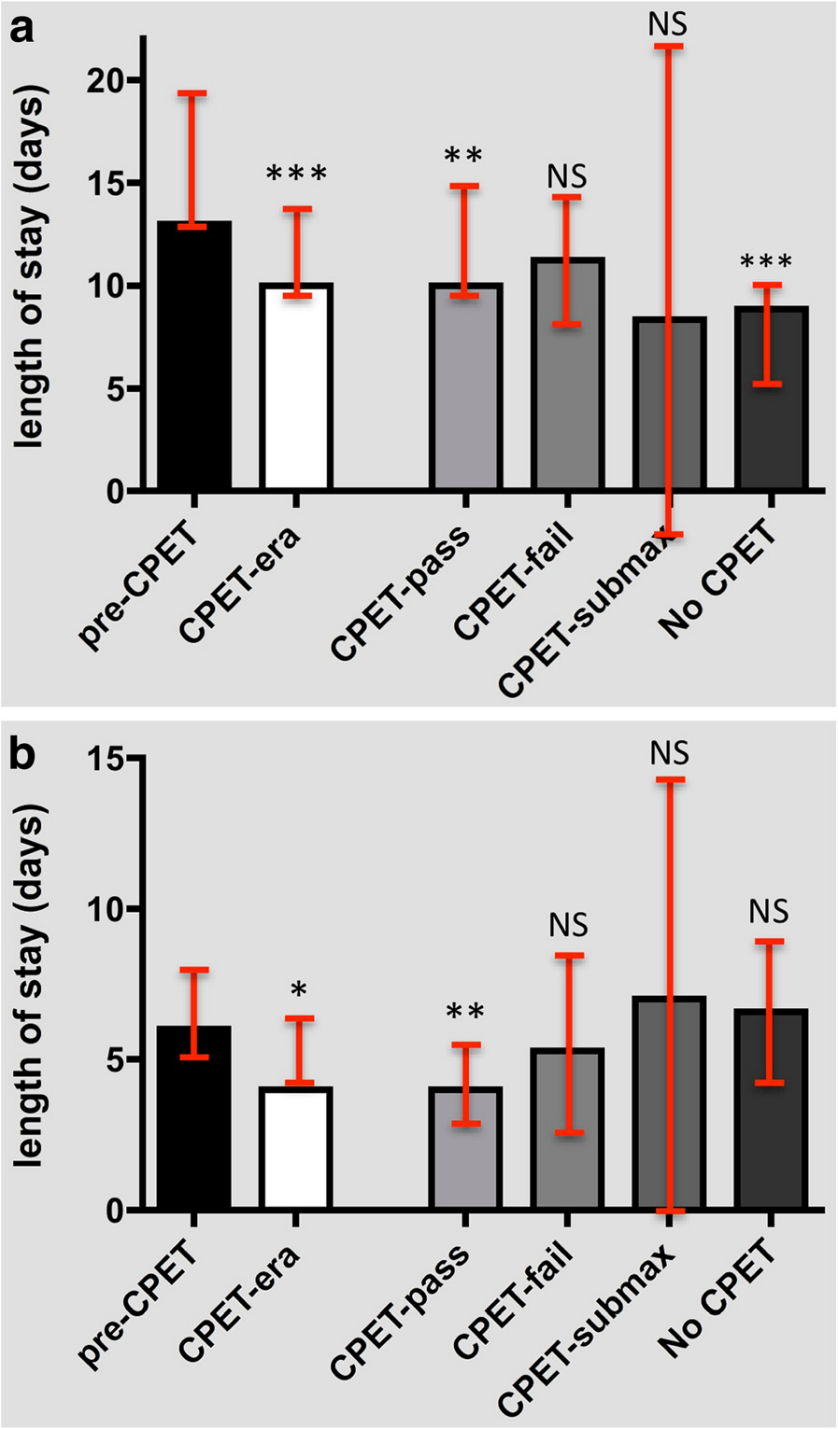
**Length of inpatient stay.** (**a**) Mann–Whitney *U* analysis of total (median) length of inpatient stay for open AAA patients in the pre- and post-CPET eras. The four bars on the right represent CPET-stratification outcomes. **P < 0.01; ***P < 0.001. (**b**) Mann–Whitney *U* analysis of total (median) length of inpatient stay for EVAR patients in the pre- and post-CPET eras. The four bars on the right represent CPET-stratification outcomes. *P < 0.05; **P < 0.01. CPET: cardiopulmonary exercise testing; EVAR: endovascular aneurysm repair; NS: not significant.

The length of inpatient stay following open AAA surgery in the CPET era (median: 10 days, 95% CI: 10.3 to 13.5) was shorter than that in the pre-CPET era (median: 13 days, 95% CI: 13.9 to 19.0; *P* < 0.001) principally due to the reduced duration of stay seen in the CPET-pass subgroup (Table 3, Figure 2a).

**Table 3:** **Mann–Whitney *U *** **comparison of length of stay for open and endovascular AAA repairs**

**Cohort**		**Median total length of stay (days) (95% CI)**	***P *** **value**	**Median length of ITU stay (days) (95% CI)**	***P *** **value**
***Open surgery***					
Pre-CPET (*n* = 103)		13 (13.9 to 19.0)		4 (5.5 to 11.2)	
CPET era (*n* = 100)	**CPET-era open (100/100)**	**10 (10.3 to 13.5)**	**P < 0.001**	**3 (3.2 to 4.4)**	**P < 0.01**
	CPET-pass (74/100)	10 (10.6 to 14.9)	***P *** **< 0.01**	3 (2.9 to 4.3)	***P *** **< 0.001**
	CPET-fail (8/100)	11.5 (8.6 to 3.9)	*P* = 0.25	4.95 (2.1 to 8.4)	*P* = 0.88
	CPET-submaximal (3/100)	11 (−5.4 to 22.0)	*P* = 0.18	11 (−5.3 to 22.0)	*P* = 0.59
	No-CPET (15/100)	8 (6.6 to 11.1)	*P* < 0.001	5 (3.1 to 6.1)	*P* = 0.82
***EVAR***^a^					
Pre-CPET (*n* = 25)		6 (5.3 to 8.6)		N/A	N/A
CPET era (*n* = 69)	**CPET-era EVAR (69/69)**	**4 (4.6 to 6.7)**	**P < 0.05**	**N/A**	**N/A**
	CPET-pass (29/69)	4 (3.6 to 5.7)	***P *** **< 0.05**	N/A	N/A
	CPET-fail (7/69)	4 (2.5 to 8.1)	*P* = 0.23	N/A	N/A
	CPET-submaximal (6/69)	4 (0 to 14.3)	*P* = 0.56	N/A	N/A
	No-CPET (27/69)	4 (4.4 to 8.8)	*P* = 0.14	N/A	N/A

^a^ Insufficient EVAR patients required ITU management for reasonable comparison.

#### EVAR

The length of inpatient stay following EVAR in the CPET era (median: 4.0 days, 95% CI: 4.6 to 6.7) was shorter than that in the pre-CPET era (median: 6.0 days, 95% CI: 5.3 to 8.6; *P* < 0.05), due to the reduced duration of stay seen in the CPET-pass subgroup (Table 3, Figure 2b).

### Duration of ITU stay

#### Open surgery

The length of ITU stay was reduced in the CPET era compared to pre-CPET controls (CPET era: 3 days, 95% CI: 3.2 to 4.4, pre-CPET: 4 days, 95% CI: 5.5 to 11.2; *P* < 0.01), reflected only by the CPET-pass subgroup (Table 3).

#### EVAR

Too few EVAR patients required ITU care in the pre-CPET or CPET-era groups for meaningful statistical comparison.

### Total non-operative inpatient costs

#### Open surgery

**Figure 3:**
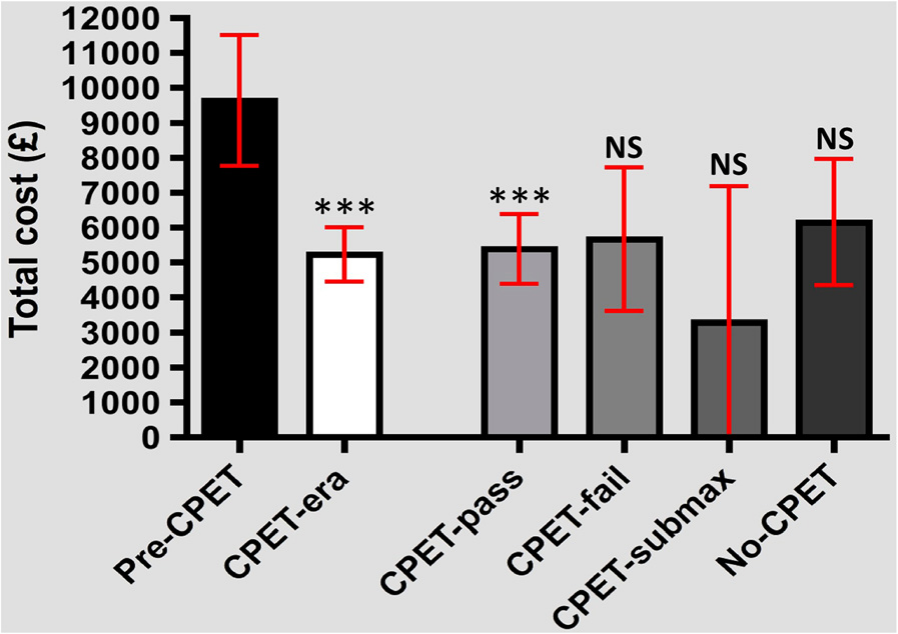
**One-way ANOVA of total cost of inpatient stay for open AAA repairs in the pre-CPET and CPET eras.** The four bars on the right represent the CPET-outcome breakdown for patients from November 2007 to the present. AAA: abdominal aortic aneurysm; ANOVA: analysis of variance; CPET: cardiopulmonary exercise testing; NS: not significant (in comparison to the median value for the pre-CPET cohort); ***: P < 0.001 (in comparison to the pre-CPET cohort).

Total non-operative (fully absorbed) costs of inpatient stay was significantly lower for the CPET-era cohort (mean: £5,229, 95% CI: 4,452 to 6,006; *P* < 0.001) compared with pre-CPET controls (mean: £9,637, 95% CI: 7,768 to 11,510). This trend is reflected only by the CPET-pass subgroup (mean: £5,387, 95% CI: 4,382 to 6,392; *P* < 0.001) (Figure 3). This is principally due to the reduced duration of ITU requirement (Table 3) and median number of end organs requiring support (pre-CPET: 2 organs, 95% CI 1.9 to 2.4; CPET-pass: 1 organ 95% CI 1.0 to 1.5; *P* < 0.001) in the CPET-pass subgroup.

The cost benefit is maintained when calculations are repeated using influenceable ward costs (pre-CPET: £8,203 c.f. CPET-era: £4,071; *P* < 0.001, CPET-pass subgroup: £4,068; *P* < 0.001).

#### EVAR

Non-operative costs are considered a function of total length of stay due to the minimal requirement for ITU in both the pre-CPET and CPET-era cohorts (Table 3).

### Total 30-day mortality

#### Open surgery

Total 30-day mortality for elective open surgery in the pre-CPET era was significantly higher than following the introduction of CPET stratification (pre-CPET 30-day mortality: 12.6%, post-CPET 30-day mortality: 4.0%; *P* < 0.05). These findings are reflected only by those in the CPET-pass subgroup (Table 4).

**Table 4:** Fisher’s exact test comparison of total 30-day mortality

**Cohort**		**30-day mortality (%)**	**Odds ratio (95% CI)**	***P *** **value**
	***Open surgery***			
**Pre-CPET** (Jan 03 to Oct 07)	Pre-CPET (*n* = 103)	12.6		
**CPET era** (Nov 07 to Jul 11)	CPET era (total) (*n* = 100)	4.0	**0.29** (0.09 to 0.92)	***P *** **< 0.05**
	CPET-pass (74/100)	2.7	**0.19** (0.04 to 0.88)	***P *** **< 0.05**
	CPET-fail (8/100)	12.5	0.989 (0.11 to 8.70)	*P* = 1.00
	CPET-submaximal (3/100)	33.3	2.31 (0.22 to 23.90)	*P* = 0.43
	No-CPET (15/100)	0	0.18 (0.01 to 3.20)	*P* = 0.21

#### EVAR

No significant difference was demonstrated in 30-day mortality following endovascular repair between the pre-CPET and CPET-era cohorts (pre-CPET 30-day mortality: 0%, CPET-era 30-day mortality: 1.4%; *P* = 1.00).

### Survival

**Figure 4:**
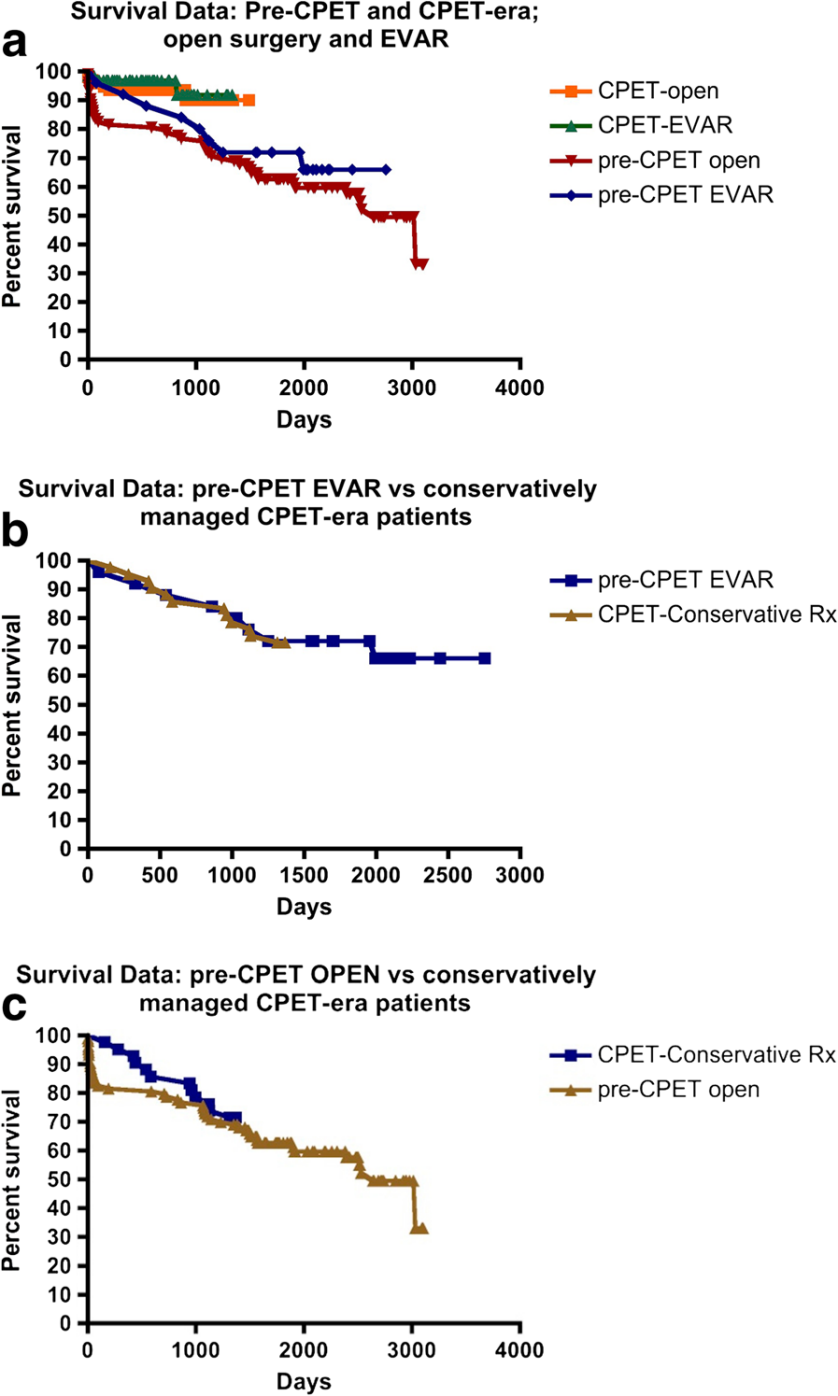
**Kaplan–Meier survival analysis (all-cause mortality).** (**a**) Comparison of open AAA repair and EVAR in the pre-CPET and CPET eras. (**b**) Comparison of pre-CPET EVAR subjects compared with CPET-era patients managed conservatively. Curve comparison by the logrank test demonstrated no significant difference (*P* = 0.96). Patients on the waiting list for aortic intervention (open or endovascular) at the close of the study have been removed from the analysis. (**c**) Comparison of pre-CPET open AAA repair compared with CPET-era patients managed conservatively. Curve comparison by logrank test demonstrated no significant difference (*P* = 0.62). Patients on the waiting list for aortic intervention (open or endovascular) at the closing date of the study have been removed from the analysis. AAA: abdominal aortic aneurysm; CPET: cardiopulmonary exercise testing; EVAR: endovascular aneurysm repair; Rx: management/treatment.

Kaplan–Meier survival analysis demonstrated reduced mid-term survival (from surgery, or from CPET for conservatively managed patients, to censorship at closure of the study) for pre-CPET EVAR and open AAA repair patients (logrank test: *P* < 0.05; Figure 4a) compared to the respective operated cohorts following CPET stratification. Median survival for pre-CPET era open AAA repairs was 2,640 days (7.23 years); however, median survival of the other subgroups could not be calculated for the period studied. The mortality-rate trends were confirmed by linear regression and remained significant (*P* < 0.001).

Survival analysis comparison between pre-CPET EVAR patients and CPET-stratified conservatively managed individuals showed no significant difference (Figure 4b) at 45 months (logrank test: *P* = 0.96).

Similarly, survival analysis comparison between pre-CPET open AAA repair patients and CPET-stratified conservatively managed individuals did not show significance (Figure 4c) at 45 months (logrank test: *P* = 0.62).

### Non-operated patients (CPET era)

**Figure 5:**
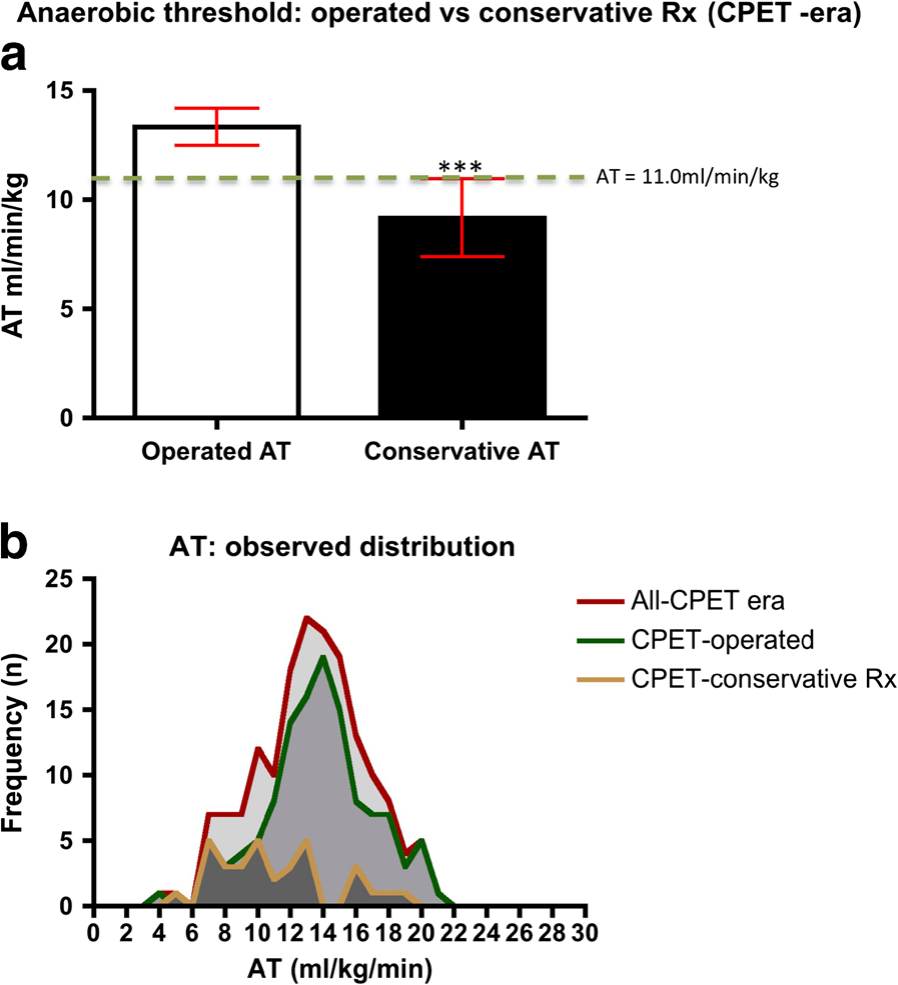
**Anaerobic threshold.** (**a)** Mann–Whitney *U* comparison of mean (95% CI) for CPET stratified and subsequently operated patients (13.3 ml/kg/min, 95% CI: 12.5 to 14.2) with those managed conservatively (9.2 ml/kg/min, 95% CI: 7.2 to 10.9;*** *P* < 0.001). The dashed line delineates the evidence-based threshold of 11.0 ml/kg/min. **(b)** Anaerobic threshold distributions for all CPET patients (*n* = 166, normal distribution on D’Agostino and Pearson omnibus normality test), CPET-era operated (*n* = 118, skewed distribution) and CPET conservatively managed subjects (*n* = 33, skewed distribution). Submaximal test results (no AT) have been removed from this analysis. AT: anaerobic threshold; CPET: cardiopulmonary exercise testing; Rx: management/treatment.

Of the 230 patients identified with elective infra-renal AAA following the introduction of CPET at UHCW NHS Trust, 61 (26.5%) had not undergone surgery prior to closure of the study. Within this group, 19 subjects were pending open or endovascular intervention (that is, on the waiting list) and 42 (18.3%) were managed conservatively. Conservative management was principally in respect of failed or submaximal CPET, reflected by the significantly lower mean AT for this cohort (Figure 5a) despite the normal distribution of AT for the entire 2007 to 2011 cohort (Figure 5b). However, in some cases, alternate non-cardiorespiratory co-morbidities (e.g. ongoing malignancy), poor quality of life or loss of independence were quoted as indications for non-operative treatment. Two individuals chose to be managed conservatively despite an adequate AT for open intervention (Figure 1).

**Figure 6:**
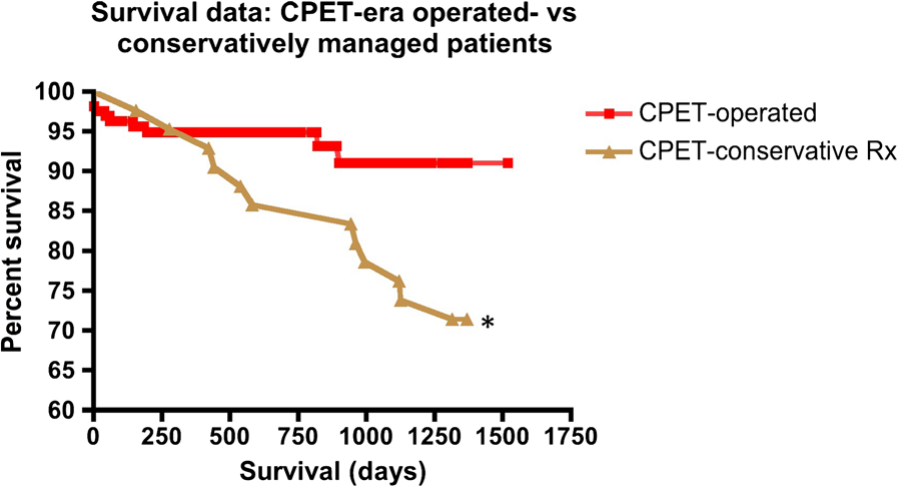
**Kaplan–Meier survival analysis (all-cause mortality) for conservatively managed patients in the CPET era in comparison to those who underwent open or endovascular surgery.** *P < 0.05; curve comparison. CPET: cardiopulmonary exercise testing; Rx: management/treatment.

Conservatively managed individuals from the CPET era exhibited comparable all-cause mortality (28.6%) to the unstratified pre-CPET control group (41.4%; *P* = 0.15). However, all-cause mortality for operated patients in the CPET era was significantly lower than those treated conservatively. These findings hold true for open and endovascular repair and were reflected only in CPET-pass patients on subgroup analysis (Table 5). A summary of all-cause mortality for conservatively managed patients is shown in Table 6. This reduction in all-cause mortality translates into a significant survival advantage for CPET-stratified operated patients compared to conservatively managed individuals over the studied period (Figure 6; logrank test (curve comparison): *P* < 0.05).

**Table 5:** Fisher’s exact test comparison of all-cause mortality

**Cohort**	**Group**	**All-cause deaths**	***P *** **value**	**Odds ratio**
**CPET era**	Conservative Rx (*n* = 42)	12 (28.6%)		
**Pre-CPET**	All (*n* = 128)	53	0.15	N/A
	Open (*n* = 103)	45	0.10	N/A
	EVAR (*n* = 25)	8	0.79	N/A
**CPET era**	All operated (*n* = 169)	11 (6.5%)	<0.001	0.17 (0.07 to 0.43)
	All open (*n* = 100)	8 (8%)	<0.01	0.22 (0.08 to 0.58)
	CPET-pass OPEN (*n* = 74)	6 (8.1%)	<0.01	0.22 (0.08 to 0.64)
	All EVAR (*n* = 69)	3 (4.3%)	<0.001	0.11 (0.03 to 0.43)
	CPET-pass EVAR (*n* = 25)	0 (0%)	<0.01	0.05 (0.005 to 0.85 )

CPET: cardiopulmonary exercise testing; EVAR: endovascular aneurysm repair; Rx: management/treatment.

**Table 6:** A summary of all-cause mortality in non-operated patients following CPET stratification

**Cohort**	**Group**	**Deaths**	**Frequency**
CPET era (*n* = 230)	Conservatively managed (*n* = 42)	Total	12 (28.6%)
		Community deaths (unknown cause)	6 (14.3%)
		Ruptured AAA	1 (2.4%)
		Cardiorespiratory disease	1 (2.4%)
		Malignancy	2 (4.8%)
		Sepsis	2 (4.8%)

AAA: abdominal aortic aneurysm; CPET: cardiopulmonary exercise testing.

## Discussion

Since the advent of CPET stratification for elective AAA patients at UHCW, a significant reduction in 30-day perioperative mortality rate (4%; Table 4) for all open repairs (*n* = 100) has been noted. Individuals achieving AT ≥ 11 ml/kg/min upon CPET who subsequently underwent open AAA repair (*n* = 74/100 open repairs) exhibited 2.7% perioperative mortality, exceeding the targets of the VSGBI QIF for 2013 [31] and this was a statistically significant reduction when compared to pre-CPET controls. By comparison, patients with AT < 11 who proceeded nonetheless to open repair (*n* = 8/100) experienced equivalent 30-day mortality (12.5%; *P* = 1.00) to the unstratified pre-CPET cohort. Individuals undergoing open AAA repair despite being unable to generate AT during CPET (*n* = 3/100) witnessed 33.3% perioperative mortality (*P* = 0.43 when compared to the pre-CPET era cohort). No perioperative deaths were reported following open AAA repair among the 15 individuals who were submitted for surgery without CPET; however this did not achieve statistical significance (*P* = 0.21) when compared to pre-CPET era controls.

A pre-operative AT ≥ 11 ml/kg/min was also associated with reduced total LOS following open repair (*P* < 0.01) and EVAR (*P* < 0.05). Of interest, the 15 individuals submitted for open surgery without CPET also demonstrated a significant reduction in total LOS (*P* < 0.001) when compared to pre-CPET controls. Conversely, the 27 individuals who underwent EVAR without CPET risk stratification did not show such a reduction in LOS (*P* = 0.14).

Following open surgery, the CPET-pass subgroup also benefited from reduced ITU LOS (*P* < 0.001) and median number of end organs supported (*P* < 0.001), which were not observed among individuals with AT < 11, no AT, or those who were not referred for CPET.

The reduction in length of inpatient stay demonstrated for both open and endovascular AAA repair may have considerable beneficial financial implications for trusts offering pre-operative CPET stratification.

EVAR patients exhibited a reduction in median length of stay from 6 to 4 days following the introduction of testing; hence there was a proportionate reduction in non-operative costs attributable to fewer bed-nights on general surgical wards. Confounding factors in this analysis include a shift from pre-discharge (inpatient) computed tomography assessment of stent-graft position prior to 2007, to 30-day surveillance as an outpatient in more recent times. This may contribute to the reduction in inpatient stay witnessed within this cohort, although of note, the reduction in bed-nights required was only significant for individuals with AT ≥ 11 on subgroup analysis. EVAR was associated with significantly shorter durations of inpatient stay, an almost abolished requirement for critical care services and lower mortality than open surgery, consistent with established work [30,36,37]. Thus, the witnessed increase in the proportion of EVAR cases should intuitively reduce overall elective AAA costs. However, the high cost of technologically advanced endovascular stents for such cases effectively abrogates this benefit when compared to open AAA repairs for individuals of adequate cardiorespiratory fitness [30,38].

A marked financial benefit was seen in open AAA repairs. Non-operative (fully absorbed) inpatient costs for the CPET-pass subgroup were approximately half of those for the pre-CPET era; there was an average saving of over £4,000 per patient. The most influential factors in this calculation included the significant reduction in ITU bed-nights utilized by AT ≥ 11 patients in addition to a lower (median) number of end organs requiring support, thereby reducing the nightly critical care tariff. Thus, pre-operative CPET risk stratification appears to be a financially advantageous method for improving perioperative outcomes in elective aortic surgery. The non-operative cost savings in open AAA surgery alone may allow generation of revenue from such testing in the longer term. An efficient CPET service should be readily achievable within the modern vascular unit; this technology having been successfully transported, assembled and utilized in a field laboratory on the South Col of Mt. Everest [39], 8,000 m above sea level.

All-cause mortality for the CPET-era cohort (2007 to 2011) was shown to be greater among individuals managed conservatively (28.6%; Tables 5 and 6) following CPET risk stratification, when compared to individuals concurrently submitted for open or endovascular surgery (6.5%; *P* < 0.001). A significant survival advantage was confirmed for surgically managed patients compared with those treated conservatively (for the period studied) according to Kaplan–Meier analysis (Figure 6). Individuals within this subgroup were shown to have poorer cardiorespiratory fitness than those within the operative subgroups (Figure 5a, mean AT 9.2 ml/kg/min c.f. 13.3 ml/kg/min; *P* < 0.001). Data for the conservatively treated subgroup (Table 6) suggest mortality predominantly resulted from significant underlying co-morbidities and supports a non-operative approach, consistent with the findings of previous studies [40]. Known mortality from ruptured AAA for the conservatively managed subgroup was 2.4%, within the limitations of this retrospective study. By implication, therefore, only a minority of deaths within this subgroup could have been prevented by intervention (open or endovascular AAA repair). However, for an individual of poor cardiorespiratory status, such an intervention would have been associated with a high risk of perioperative complications and mortality [36,40]; that is, death or serious morbidity may have been hastened by surgery. Longer-term follow-up would be required, ideally within the confines of a prospective study, to ascertain the natural progression of CPET-stratified conservatively managed patients. Assessment of the frequency of AAA-related and other deaths, morbidity and survival may develop a clearer evidence base for the optimum management of unfit individuals.

A remaining equivocation relates to the degree of stringency with which pre-operative CPET stratification is applied as a gateway to open surgery. The findings of this study show that a dramatic reduction in 30-day mortality (12.6% to 2.7%) is achievable if an AT ≥ 11.0 is considered an absolute requirement. By contrast CPET-guided pre-operative decision-making (allowing for clinical discretion) resulted in 4% 30-day mortality and permitted surgical management of a further 26 individuals. Ultimately, individual vascular units offering this service will have to choose between optimizing perioperative mortality, or allowing patients of borderline cardiorespiratory fitness the chance of an elective AAA repair. This controversy, perhaps, warrants high-level debate as we move towards the 2013 target of 3.5% 30-day mortality, as set by the VSGBI QIF [31].

Patients undergoing open or endovascular AAA repair in the pre-CPET era exhibited reduced mid-term survival when compared to the CPET-era operated cohorts. Indeed, at 45 months no significant survival advantage was conferred to the unstratified open surgery or EVAR patients, when compared to CPET-stratified conservatively managed individuals (Figure 4b,c). For pre-CPET EVAR patients, who demonstrated negligible perioperative mortality rates, a prevalence of significant underlying co-morbidities in subjects selected for this intervention may contribute to these data, consistent with the findings of Goodney *et al.*[41]. The less metabolically demanding EVAR is routinely offered to carefully selected patients who fail to achieve a satisfactory AT on exercise testing. Equivalent survival between these patients and conservatively managed individuals, again, suggests a requirement for further study; conservative management may be more appropriate for such patients [40], based upon specific morbidities, quality-of-life outcomes and mode of death.

For pre-CPET-era open AAA repairs, the finding of equivalent survival at 45 months (Figure 4c) when compared to CPET-stratified individuals rejected for surgery, strongly reflects the perioperative death rate. This is suggested by the significantly reduced perioperative mortality rates for CPET-stratified open AAA patients and the concurrent mid-term survival advantage seen for this cohort (Figure 4a). Moreover, the improved survival trend for CPET-stratified open AAA patients suggests that their superior cardiorespiratory fitness (Figure 5a) may preserve the survival advantage in the longer term and should be subject to further study.

### Limitations of the study

Compliance with the trust-wide guideline of pre-operative CPET for all elective AAA patients was incomplete, with 42 (18.3%) patients submitted for surgery (15 open repairs and 27 EVAR) without testing. Moreover, an element of cross-over was permitted following risk stratification; individuals within the CPET-fail (AT < 11) or CPET-submaximal (no AT) subgroups were not prevented from proceeding to open surgery upon request. A number of patients were disinclined to manage their AAA expectantly (when consideration was given to the accepted 90% mortality following rupture [32]), instead they accepted the increased risk of perioperative mortality and proceeded to intervention. Morally, this was a difficult view to oppose. Such limitations of this retrospective study dictated a need for subgroup analysis of the open repair and EVAR patient groups within the CPET-era cohort (see Figure 1) to avoid confounding results.

Furthermore, despite exhaustive efforts to identify all patients with elective infra-renal AAA within both cohorts, no conservatively managed individuals could be detected in the pre-CPET era. Notwithstanding the trust’s all-comers policy to aneurysm repair prior to 2007, the authors are reluctant to accept that there were no such patients. By implication, a degree of misclassification and data loss has to be assumed (leading to information bias). Such bias is compensated in part, by the subgroup analyses performed. However, potentially valuable comparisons between subjectively determined conservatively managed individuals of the pre-CPET era and those objectively identified by CPET, were not possible.

## Conclusion

The introduction of pre-operative CPET risk stratification for elective AAA repair patients (as a quality improvement strategy) has shown improved perioperative outcomes.

In this retrospective study, an anaerobic threshold of ≥ 11.0 ml/kg/min has been positively associated with reduced perioperative (30-day) mortality, total LOS, length of ITU stay and support requirements for open surgical patients. As a consequence, non-operative costs were significantly reduced for these individuals. For EVAR patients, AT ≥ 11.0 was similarly associated with a reduced total LOS and thus, non-operative costs.

A significant mid-term survival advantage is also seen for CPET-stratified open repair and EVAR cohorts over controls, consistent with previous findings [26]. A consequence of CPET stratification (and clinical discretion) was the generation of a conservatively managed, unfit patient cohort. These individuals demonstrated greater all-cause mortality than surgically managed patients, principally of non-aneurysmal etiology, justifying the non-operative approach.

### Recommendations for further study

A prospective randomized controlled trial (RCT) would be scientifically most appropriate to confirm the findings of this study, potentially implicating CPET as an appropriate risk assessment tool contributing to mortality prediction in AAA surgery, as per NICE recommendations for further research [42]. A recently published study suggests that the paucity of robust data should preclude routine adoption of CPET in risk-stratifying patients undergoing major vascular surgery [15]. However, to the authors’ knowledge, this manuscript describes the largest current single-center UK series of patients risk-stratified in this manner for elective AAA repair, with numerous potentially advantageous outcomes. Thus, there is precedent for a RCT to clarify this ongoing and controversial issue.

## Abbreviations

AAA: Abdominal aortic aneurysm; ANOVA: Analysis of variance; AT: Anaerobic threshold; BMI: Body mass index; CPET: Cardiopulmonary exercise testing; CRRS: Clinical results reporting system; ECG: Electrocardiogram; EVAR: Endovascular aneurysm repair; FEV1: Forced expiratory volume in 1 second; FVC: Forced vital capacity; LOS: Length of inpatient stay; ITU: Intensive therapy unit; MET: Metabolic equivalent; NHS: National Health Service; NICE: National Institute for Health and Care Excellence; NS: Not significant; QIF: Quality improvement framework; RCT: Randomized controlled trial; Rx: Management/treatment; UHCW: University Hospitals Coventry and Warwickshire; VSGBI: Vascular Society of Great Britain and Ireland.

## Competing interests

The authors declare that they have no competing interests.

## Authors’ contributions

SJG: data collection, interpretation, analysis, drafting and revision of manuscript, intellectual content of study. HY: concept and design, data collection. MS: data collection and analysis. JS: data collection, drafting of manuscript, critical revisions. CH: data collection. DW: data analysis, interpretation, critical revisions for intellectual content. CM: revision of manuscript, intellectual content. AM: revision of manuscript, intellectual content. DH: concept, intellectual content. CHEI: concept and design, data interpretation, drafting and revision of manuscript, intellectual content of study. All authors read and approved the final manuscript.

## Authors’ information

SJG: MBChB MD MRCS. SpR in Vascular/General Surgery, West Midlands Deanery.

HY: MBChB FRCS. SpR in Vascular/General Surgery, West Midlands Deanery.

MS: MBChB MRCS. Research Registrar in Vascular Surgery, UHCW NHS Trust.

JS: MSc BSc. Clinical Service Manager, Department of Respiratory Physiology, University Hospitals Coventry and Warwickshire NHS Trust.

CEH: MBChB MRCS. SpR in Trauma and Orthopedics. West Midlands Deanery.

DW: FRCA. Consultant in Critical Care and Anesthetics. Medical Lead Central England Critical Care Network and Chair of the National Forum of Medical Leads for Critical Care Networks.

CM: MBBS FRCS. Consultant Vascular Surgeon. UHCW NHS Trust.

AM: MD FRCS. Consultant Vascular and Endovascular Surgeon, UHCW NHS Trust.

DH: MS MMedEd FRCS. Consultant Vascular Surgeon and Clinical Lead, UHCW NHS Trust.

CHEI: (Professor) PhD FRCS FRCP. Director of Research and Development, Associate Medical Director. Consultant Vascular, Endovascular and Renal Transplant Surgeon

Warwick Medical School and University Hospitals Coventry and Warwickshire NHS Trust.

## Collaborators

Andrew Taylor (Finance Manager, Surgical Directorate, UHCW NHS Trust), Andrew Roberts (Information Systems Manager Department of Critical Care, UHCW NHS Trust), Julie Aughton (Specialist Respiratory Physiologist), Mr P Blacklay and Mr K Zayyan (Consultant Vascular Surgeons), Dr A Scase, Dr A Thacker, Dr E Borman, Dr B Dudkovsky, Dr S Sreevathsa and Dr A Kelly (Consultant Anesthetists).
